# Design of Nickel-Containing Nanocomposites Based on Ordered Mesoporous Silica: Synthesis, Structure, and Methylene Blue Adsorption

**DOI:** 10.3390/gels10020133

**Published:** 2024-02-06

**Authors:** Tatyana Kouznetsova, Andrei Ivanets, Vladimir Prozorovich, Polina Shornikova, Lizaveta Kapysh, Qiang Tian, László Péter, László Trif, László Almásy

**Affiliations:** 1 Institute of General and Inorganic Chemistry, NAS of Belarus, 220012 Minsk, Belarus; tatyana.fk@gmail.com (T.K.); andreiivanets@yandex.ru (A.I.);; 2 State Key Laboratory of Environment-Friendly Energy Materials, Southwest University of Science and Technology, Mianyang 621010, China; 3 Institute for Solid State Physics and Optics, HUN-REN Wigner Research Centre for Physics, Konkoly Thege Miklós str. 29-33, 1121 Budapest, Hungary; peter.laszlo@wigner.hun-ren.hu; 4 Institute for Energy Security and Environmental Safety, HUN-REN Research Centre for Natural Sciences, Konkoly Thege Miklós str. 29-33, 1121 Budapest, Hungary; trif.laszlo@ttk.hu; 5 Institute for Energy Security and Environmental Safety, HUN-REN Centre for Energy Research, Konkoly Thege Miklós str. 29-33, 1121 Budapest, Hungary

**Keywords:** sol-gel, mesoporous silica, adsorption, heteroatom, Ni, small-angle neutron scattering, methylene blue

## Abstract

Mesoporous materials containing heteroelements have a huge potential for use as catalysts, exchangers, and adsorbents due to their tunable nanometer-sized pores and exceptionally large internal surfaces accessible to bulky organic molecules. In the present work, ordered mesoporous silica containing Ni atoms as active sites was synthesized by a new low-temperature method of condensation of silica precursors on a micellar template from aqueous solutions in the presence of nickel salt. The homogeneity of the resulting product was achieved by introducing ammonia and ammonium salt as a buffer to maintain a constant pH value. The obtained materials were characterized by nitrogen sorption, X-ray and neutron diffraction, scanning electron microscopy, infrared spectroscopy, and thermal analysis. Their morphology consists of polydisperse spherical particles 50–300 nm in size, with a hexagonally ordered channel structure, high specific surface area (A_BET_ = 900–1200 m^2^/g), large pore volume (V_p_ = 0.70–0.90 cm^3^/g), average mesopore diameter of about 3 nm, and narrow pore size distribution. Adsorption tests for methylene blue show sorption capacities reaching 39–42 mg/g at alkaline pH. The advantages of producing nickel silicates by this method, in contrast to precipitation from silicon alkoxides, are the low cost of reagents, fire safety, room-temperature processing, and the absence of specific problems associated with the use of ethanol as a solvent, as well as the absence of the inevitable capture of organic matter in the precipitation process.

## 1. Introduction

Nanomaterials have emerged as a novel class of materials with at least one dimension in the range from 1 to 100 nm. Their macroscopic properties can be tuned as desired by controlling the size, shape, synthesis conditions, and functionalization. Exceptionally high surface areas can be achieved through the rational design of nanomaterials [1,2]. Porous materials with a high specific surface area and nanostructured morphology are in great demand in adsorption, separation, heterogeneous catalysis, gas storage, and sensor technologies, and they are promising for creating high-tech and science-intensive industries. Strategies for their production, main properties, and applications are widely described in a number of research studies and reviews [1,2,3,4,5,6,7]. Research and development of ordered mesoporous materials with uniform pores, unique topology, controlled acidity, and high structural stability for new scientific and technological applications are increasingly attracting the attention of researchers. Sufficiently wide pores of mesoporous catalysts and carriers in industrial technologies make it possible to minimize the problems of mass transfer of reactants and products inherent in zeolites. Many papers report on the advantageous properties related to the regular morphology, i.e., ordered matrix of mesopores, which affects the adsorption and catalytic activity [4,5,8].

After the discovery of mesoporous molecular sieves, a large number of studies [9,10,11,12] have been devoted to these materials, which can be widely used in various fields depending on the conditions of their synthesis and chemical design [13,14,15,16,17]. However, due to the limited number of active sites on the pure silica surface of the zeolite, its use in chemical reactions is limited. Hence, it becomes necessary to include active sites in the framework of zeolite-like materials in order to expand their application in adsorption, catalysis and other fields [9]. Many studies in recent years have shown that heteroatoms such as Fe, Cr, V, and Ni in molecular sieves increase their potential as adsorbents, catalysts, and ion exchangers and, due to the rearrangement of nanosized pore diameters, increase their exceptionally high surface area and availability of sites accessible to bulky organic molecules [18,19,20].

Conventional impregnation and ion exchange methods do not guarantee that heteroatoms can become an integral part of zeolite-like materials. It is also considered difficult to increase the content of specific heteroatoms to more than 5–8 wt% by directly adding a metal-containing precursor compound to the gel mixture [21,22,23,24]. However, if the production method for high-transition metal molecular sieves is improved, it is expected that ordered mesoporous materials will be able to expand the field of both adsorption and catalytic applications [25].

In the majority of works, mesoporous silica and metal–silicate materials are prepared using the sol-gel process with tetraethyl orthosilicate (TEOS), or other alkoxy silicates as the silicon source, e.g., in the latest paper of Cho et al. [26], by adding metal salts to aqueous alcoholic TEOS solutions, and many other similar studies [27,28,29,30]. For practical applications and large-scale use, the relatively expensive TEOS has to be substituted by alternative silicon sources, and alcohol-free synthesis methods are also advantageous.

In this work, we synthesized ordered mesoporous silicas using a low-cost method with sodium metasilicate, Na_2_SiO_3_·9H_2_O, and modifying in situ with a transition metal using nickel as an example. Different Ni/Si reagent molar ratios were used in order to expand the range of heteroatom content in the anionic silica framework which can influence and improve the adsorption and catalytic properties of these mesostructured silicate materials. The synthesis of silica modified with nickel in situ was carried out at a constant molar ratio of SiO_2_ and cetylpyridinium chloride and at various Ni/Si molar ratios and at three different pH values. Chemically pure nickel chloride, NiCl_2_·6H_2_O, and sodium metasilicate, Na_2_SiO_3_·9H_2_O, with a SiO_2_ content of 5.5 mass %, were used as starting materials, and the sol-gel reaction was carried out at room temperature. In addition, in this work, we considered the traditional issue of environmental remediation and used the resulting porous materials as adsorbents of harmful pollutants from water on the example of methylene blue dye [31,32,33,34]. The obtained results demonstrate the applicability of the proposed approach to obtain efficient inorganic sorbents for the sorptive removal of organic pollutants.

## 2. Results and Discussion

### 2.1. Nitrogen Adsorption–Desorption

**Figure 1:**
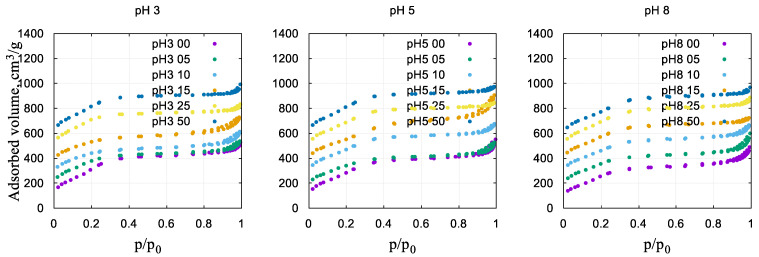
Nitrogen adsorption–desorption isotherms of Ni–silicate composites. Isotherm data are shifted vertically.

Samples of nickel silicates are typical mesoporous materials exhibiting various types of capillary condensation at intermediate relative pressures. The experimental adsorption–desorption isotherms are shown in Figure 1. The isotherm shapes indicate that all samples are typical mesoporous materials exhibiting capillary condensation at intermediate pressures.

The calculated characteristics are shown in Table 1. The samples are characterized by high specific surface values, reaching A_BET_ = 1200 m^2^/g, and pore volumes in the range of 0.3–0.8 cm^3^/g.

Limited diffusion of nitrogen molecules prevents them from entering the narrowest pores (D < 0.7 nm at p/p_0_ < 0.01). The surface area of the nitrogen-measurable microporous component, A_micro_, could be obtained as the difference between the specific surface area, according to the BET equation, and the external surface area, A_ext_, calculated by the t-method, assuming A_micro_ = A_BET_ − A_ext_. To achieve this, the analyzed isotherm was rearranged into a t-plot as a function n = f(t):

n_a_ = b_t_ t + b, (1)

where n_a_ is the amount of nitrogen adsorbed in equilibrium conditions; t = (n/n_m_) × σ, where n/n_m_ is the number of statistical monolayers in the film; b_t_ is the t-plot angle tangent equal to n_m_/σ; b is the segment clipped by the t-plot on the ordinate axis when extrapolated in cases where the isotherm is not identical to the standard isotherm; nm is the capacity of the monolayer in moles of adsorbate; σ is the thickness of one layer equal to 0.354 nm for nitrogen at 77 K, assuming the densest hexagonal packing of molecules in the adsorption film.

The external surface area, A_ext_, determined by the t-method, is equal to A_ext_ = b_t_ V_L_, where V_L_ is the molar volume of nitrogen at 77 K.

An increase in the Ni/Si molar ratio leads mainly to a decrease in the surface area of the samples, with slight fluctuations in its values either up or down. The inclusion of nickel in the silicate framework in the studied range of Ni/Si values, as expected, affects the morphology of the samples, destroying the ordered packing of mesopores, followed by a moderate reduction in the porosity.

**Figure 2:**
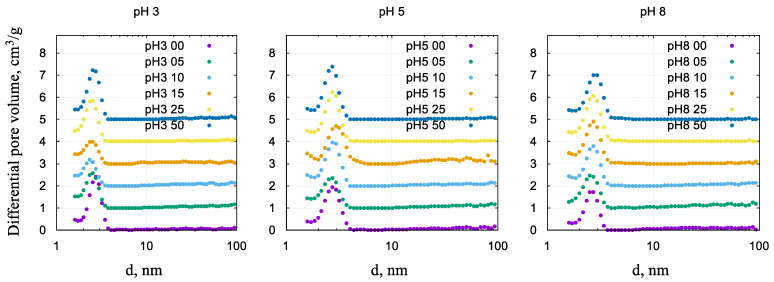
Differential curves of the NLDFT pore size distribution of nickel nanocomposites prepared in different pH conditions. The data are shifted vertically for clarity.

Figure 2 shows the non-local density functional theory (NLDFT) pore size distribution curves of samples prepared at various pH values and molar ratios of nickel and silicon. According to the distributions (Figure 2) and formal BJH calculations (Table 1), the average (hydraulic) pore diameter of the samples is in a narrow range of 2–3 nm. The pore size distribution of the Ni-free samples appears to be more narrow (bottom data set in Figure 2).

The experimental results and calculated data prove that with increasing pH, the specific values of the surface area and pore volume of nickel-containing materials somewhat decrease, while the average pore size somewhat increases. There are several mechanisms that can explain the decrease in the surface area of nickel silicates prepared at different pH values with increasing nickel content:

(1) Intrapore formation of sufficiently small nickel(II) oxide nanoparticles, which are finely dispersed inside mesopores and not detected by X-ray phase analysis;
(2) Blocking of the pores of the mesostructure with fairly bulky nickel(II) oxides;
(3) The introduction of nickel(II) ions into the silica framework, which leads to an increase in the density of the composite;
(4) A buildup of secondary and tertiary mesoporosity.

### 2.2. Scanning Electron Microscopy

**Figure 3:**
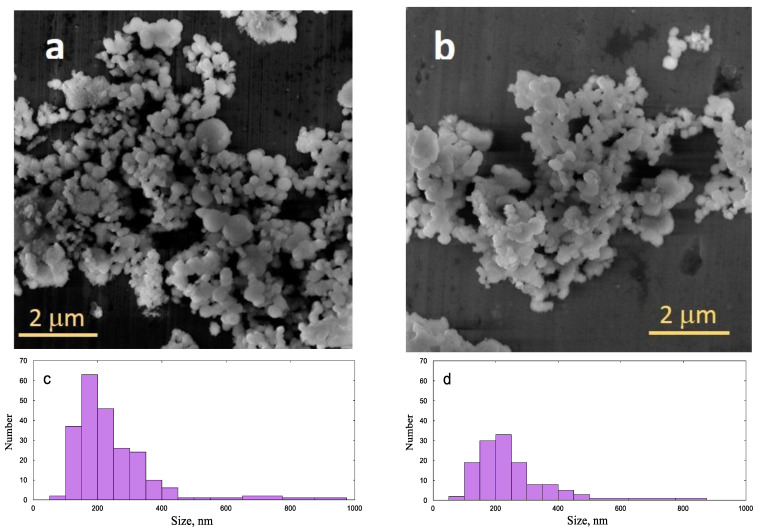
SEM images of the studied xerogels and calculated particle size distributions: (**a**,**c**) sample “pH8 10”; (**b**,**d**) sample “pH8 50”.

All studied samples reveal the typical compact particle morphology of the MCM-41-type silica, prepared in basic conditions. Selected images of samples prepared with different Ni contents are shown in Figure 3. Two characteristic morphologies can be recognized, which pertain for all samples: near-spherical particles with a smooth surface and typical size of 200–400 nm, and groups of much smaller particles, which form agglomerates of submicron size.

### 2.3. Infrared Spectroscopy

**Figure 4:**
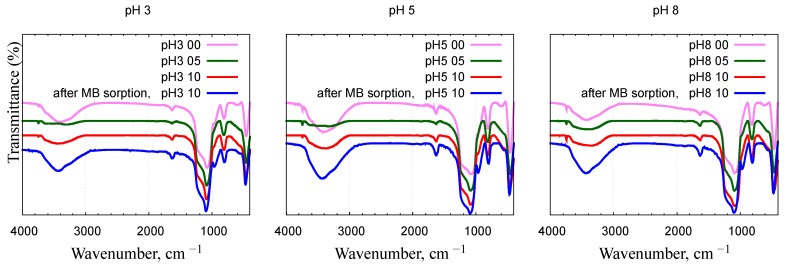
FT-IR spectra of nickel–silica nanocomposites prepared in different pH conditions. The data are shifted vertically. Spectra for samples “pH3 10”, “pH5 10”, and “pH8 10” after methylene blue sorption are shown by blue line.

The infrared (IR) spectra of the samples obtained, measured in the wave number range of 4000–400 cm^−1^, are shown in Figure 4. Surface silanol groups absorb in clearly distinct regions of the IR spectrum: approximately 3750, 3650, 3500, and 1640 cm^−1^ [35,36]. Peaks at 3340–3400 cm^–1^ correspond either to physically adsorbed water held by hydrogen bonds or by surface groups containing paired hydroxyl groups of =Si(OH)_2_. The same can be said about the 1630–1640 cm^–1^ band, since it is known that, along with physically sorbed water, this wave number corresponds to one of the harmonics of the Si-O bond. Peaks at 1630–1640 cm^−1^ correspond to deformation vibrations of O-H groups [37]. Wave numbers 1240–970, 800, and 460 cm^–1^ correspond to bands that can be attributed to various vibrations of the framework structure of silica materials [38,39]. The peak between 1250 and 900 cm^–1^ is, in all likelihood, distorted and shifted toward 1085–1073 cm^−1^.

The appearance of a shoulder at about 960–970 cm^−1^ confirms that the nickel atoms interact with the silica framework and are introduced into its structure. Absorption at 960–970 cm^−1^ refers to vibrations of those Si-O bonds in which oxygen is bound to only one silicon atom; the second valence can be compensated by a metal cation or a proton. In the calcined samples, this band appears in the IR spectra only after inclusion of Ni as a Si-O-Ni moiety into silica. The band intensity increases with increasing Ni/Si molar ratio. In hydroxylated non-calcined silica, the 960–970 cm^−1^ band corresponds to the stretching vibrations of the Si-O-H bonds. In calcined pure silica, this band is absent due to the absence of Si-O-Ni bonds.

### 2.4. X-ray Diffraction

**Figure 5:**
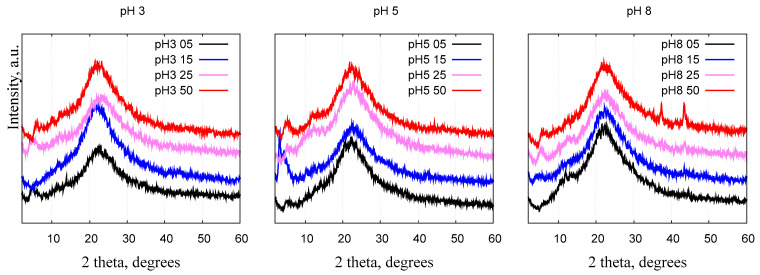
X-ray diffractograms of nickel–silica nanocomposites prepared in different pH conditions. The data are shifted vertically.

All samples show diffraction patterns characteristic to amorphous silica materials (Figure 5), a broad peak centered between 20° and 25° [40,41]. In the wide-angle region, two weak peaks emerge from the background of an amorphous silica halo which become more noticeable with an increase in the Ni/Si ratio and pH values. Sample “pH8 50” has two peaks at 37° and 43°, characteristic of the (111) and (200) reflections of the NiO phase [41,42]. Reflections in the small-angle region below 10° are related to the ordered mesoporous structure of silica and are better resolved in the small-angle scattering data (see Section 2.5).

According to the X-ray diffraction results in relation to pore ordering (small-angle range) and the possible formation of nickel oxide crystallites (wide-angle range), it can be concluded that part of the nickel is included in the silica walls, and part of the nickel can exist outside the silica framework in the form of metal oxide crystalline aggregates.

### 2.5. Small-Angle Neutron Scattering

**Figure 6:**
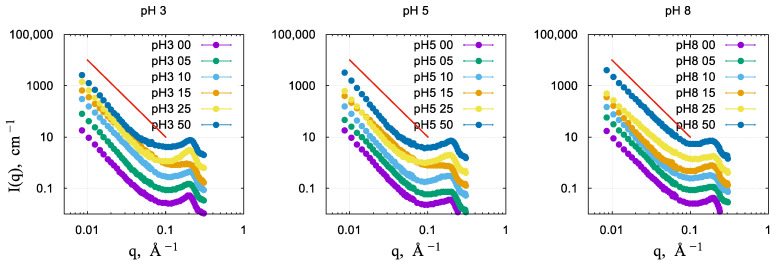
Small-angle neutron scattering data on the xerogels prepared in different conditions: pH 3, pH 5, and pH 8. The solid line shows a slope −3 in double logarithmic coordinates.

Neutron diffractograms in the small-angle range are shown in Figure 6. The diffraction peak seen at q = 0.2 Å^−1^ corresponds to the (001) reflection of the 2D hexagonal lattice of the MCM-41 materials [22,27,28,29,43,44,45,46,47]. The variation in the synthesis pH and the Si/Ni ratio results in moderate and non-regular variations in the periodicity of the material and the long-range order. The low-q part of the scattering curves shows linear behavior in the double logarithmic representation, which can point to a fractal structure of the materials or indicate a high polydispersity of the nanoparticles. The value of the exponent for all samples is close to −3, which is the border value between surface and volume fractal morphology [48]. Since the variation in the exponent value with the Ni content of the samples is not regular, the polydispersity scenario is more plausible, and it is also supported by the observations through electron microscopy. Altogether, these results show that introducing Ni atoms into the pore walls does not destroy the long-range order and the high porosity of the materials, in agreement with the results of nitrogen sorption measurements.

### 2.6. Thermal Analysis

**Figure 7:**
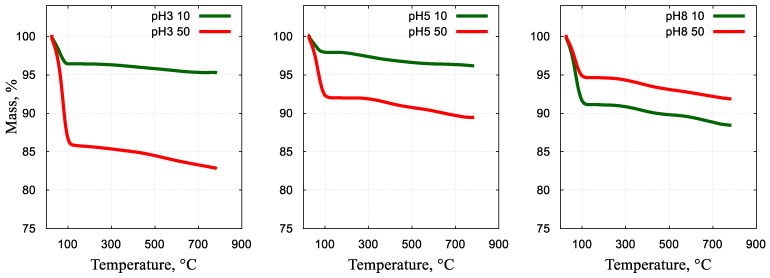
Thermal analysis curves for composites prepared with different Ni/Si ratios and in different pH conditions.

Thermogravimetric analyses in synthetic air were performed on selected samples with 10:90 and 50:50 Ni/Si ratios in order to reveal the influence of synthesis conditions on the thermal stability of the mesoporous composites. The weight loss curves for the six samples studied are shown in Figure 7. For all samples, two distinct weight loss regions can be identified, from which the first one occurs below 150 °C and corresponds to the evaporation of the surface-bound water. The amount of water lost in this region is between 2 and 14%. The further weight decrease, observable between 150 and 750 °C, can be associated with the evaporation of the chemically bound water and dehydroxylation of the silica surfaces. Also, the remnants of the organic precursor can burn and escape at temperatures higher than those applied during the calcination of the samples. Altogether, the materials look stable against degradation up to 300–500 °C.

### 2.7. Removal of Methylene Blue Dye from Aqueous Media by Nickel-Containing Nanocomposites

The prepared nanocomposites were tested for sorptive removal capacity of a widely used organic pollutant, the cationic dye methylene blue (MB), used in large amounts in industry for coloring paper, dyeing cotton, wools, silk, and leather, but used also in medication and diagnostic biology [49,50,51].

**Figure 8:**
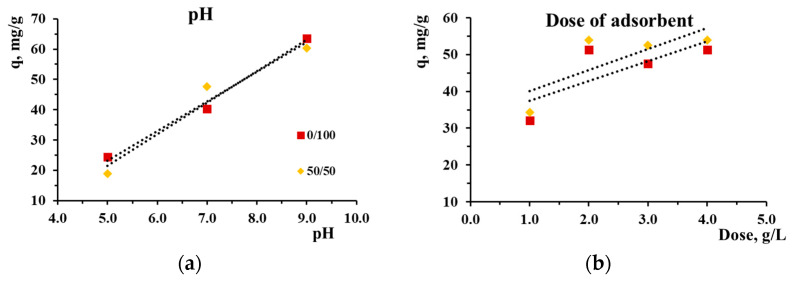
Effect of the solution pH (**a**) and adsorbent dose (**b**) on methylene blue adsorption capacity on mesoporous Ni–silica samples.

In a preliminary experiment, the optimal conditions for effective adsorption of MB by selected adsorbents were determined. The choice of adsorbents was determined by the use of undoped silica and silica doped with the maximum amount of nickel. Thus, when studying the effect of the pH of the model pollutant solution (Figure 8a) on the adsorption characteristics of the obtained adsorbents, it was found that the increase in pH was almost proportional to the increase in adsorption capacity in the range of 5.0–9.0. Similar behavior for MB sorption on silica was reported in other studies [28,32]. A further increase in pH is not justified, since it will be far from the pH of real wastewater containing MB dye [52]. Therefore, pH 9.0 was chosen as optimal for further experiments. 

At the same time, the study of the effect of the adsorbent dose (Figure 8b) on the adsorption characteristics showed that with an increase in the adsorbent dose from 1.0 to 2.0 g/L, the sorption capacity and removal efficiency increased approximately 1.6 times. A further increase in the adsorbent dose did not lead to a significant change in these adsorption characteristics. Therefore, an adsorbent dose of 2.0 g/L was used in further experiments.

The calculated sorption characteristics (sorption capacity and removal efficiency) depending on the preparation conditions of the adsorbents (pH of precipitation; Ni/Si ratio) are presented in Table 2.

Based on the data obtained (Table 1 and Table 2), it was proved that an increase in the nickel content in nickel silicates does not lead to a noticeable degradation of the mesoporous structure of adsorbents and a decrease in their sorption characteristics relative to methylene blue. The sorption capacities were found for all samples in the range 39–42 mg/g and the removal efficiency reached 91–100%, which is comparable (Table 3) to the performance of other mesoporous silica sorbents of MCM-41 type, obtained by a more complex alkoxide method and modified with transition metals [28,53,54], or significantly inferior to neat mesoporous SBA-15 silica with double-sized pores [55,56]. It can be seen that the inclusion of Ni does not influence noticeably the sorption capacity. Furthermore, the pH used in the synthesis has no obvious effect on the sorption, and the removal efficiencies are all above 90% in the chosen experimental conditions. 

Infrared spectra were taken on dried silica powders after MB adsorption; selected spectra are shown in Figure 4. No visible traces of bands associated with MB molecules can be seen. The disappearance of the free silanol vibrations at 3750 cm^−1^ testifies to the involvement of the surface hydroxyl groups in the adsorption process. The interaction between the dye and Ni-MCM-41 is mostly electrostatic, as evidenced from the pH dependence of the sorption capacity (Figure 8a). Hydrogen bonds can also form between the surface O-H groups and N atoms of MB. The attraction between the π electrons of the MB aromatic cycles and either the non-bonding electrons of the O-H groups or the Ni ions is another possible binding mechanism.

## 3. Conclusions

Silica materials were obtained using sol-gel technology on a supramolecular liquid crystal template in the presence of nickel ions. Mesoporous silica with uniform porosity and ordered nanostructural morphology was obtained. The advantages of the developed method of synthesis are the use of a low-cost silica precursor and the variation in the silica/metal ratio in a wide range. Additionally, the synthesis is feasible over a wide range of pH values, allowing for high flexibility in choosing synthesis conditions.

The obtained materials have a high BET specific surface area (A_BET_ = 900–1200 m^2^/g) and a large pore volume (V_p_ ≈ 0.70–0.90 cm^3^/g), with an average pore diameter in the mesopore range (2–3 nm) and narrow pore size distribution.

X-ray diffraction showed the overall amorphous glassy silica matrix with a detectable NiO phase for samples synthesized at high Ni content in the precursor mixture, whereas low-angle neutron diffraction revealed the hexagonally ordered structure of elongated pores, typical to MCM-41 materials, for all sample compositions and synthesis conditions.

Infrared spectroscopy and X-ray phase analysis proved that nickel atoms interact with the surface of pore channels with the formation of active surface Si-O-Ni groups.

Adsorption tests for methylene blue show sorption capacities reaching 39–42 mg/g at ambient temperature and alkaline pH. The obvious advantages of producing nickel silicates by this method, in contrast to precipitation from alkoxides, are fire safety, room temperature conditions, and the absence of specific problems associated with the use of ethanol as a solvent.

## 4. Materials and Methods

### 4.1. Synthesis of Nickel Silicate

Chemically pure nickel chloride, NiCl_2_·6H_2_O, and oligomeric species of silicon(IV) oxide in the form of sodium metasilicate, Na_2_SiO_3_·9H_2_O, with a SiO_2_ content of 5.5 mass %, were used as starting materials (Sigma Aldrich, St. Louis, MO, USA). The low-temperature synthesis of ordered mesoporous silica modified with nickel in situ was carried out by the sol-gel method at a constant molar ratio of SiO_2_ and cetylpyridinium chloride and at various Ni/Si molar ratios. Aqueous cetylpyridinium chloride in micellar form was used as a template. To neutralize the alkali, 35% sulfuric acid was used. The metal salt added to the reaction mixture was hydrolyzed by adjusting with a solution of NH_3_·H_2_O to fixed pH values 3, 5, and 8. The precipitate was separated and dried in air, and suspended in an ammonium sulfate solution and twice in an NH_3_·H_2_O solution, after which it was washed twice with distilled water and dried in air. The introduction of ammonia and ammonium salt, which also served as buffers to maintain a constant pH, increased the homogeneity of the resulting product. The xerogel was calcined in a muffle furnace at 923 K for 2 h. The list of samples with preparation conditions and Ni/Si ratios is included in Table 1.

The detailed protocol of the synthesis procedure was as follows. For example, the synthesis scheme for the sample, designated by the acronym “pH5 10” in Table 1, consisted of the following stages: (1) dissolving 2.5 g of surfactant in 47.5 g of distilled water at 313 K; (2) adding 13.01 g of Na_2_SiO_3_·9H_2_O to the resulting solution at 313 K; (3) adding 11.38 mL of 35% H_2_SO_4_ and stirring the resulting solution for 30 min at 313 K; (4) adding 1.21 g of NiCl_2_·6H_2_O and stirring the resulting solution for 30 min at 313 K; (5) increasing the pH value of the solution to 5 by adding an aqueous solution of NH_3_·H_2_O; (6) separating the precipitate and drying it at room temperature; (7) washing the xerogel with a 3% aqueous solution of (NH_4_)_2_SO_4_, NH_3_·H_2_O, and distilled water; (8) calcination of the xerogel at 923 K for 2 h.

### 4.2. Characterization

#### 4.2.1. Nitrogen Sorption

The porosity was measured by the N_2_ physisorption technique. The texture of the samples was evaluated by the specific characteristics of the pore volume and surface area calculated from low-temperature nitrogen adsorption–desorption isotherms. Isotherms were measured by the volumetric method on an ASAP 2020MP analyzer (Micromeritics Instrument Corporation, Norcross, GA, USA). Before analysis, the samples were degassed at a temperature of 523 K and a residual pressure of 1.3 × 10^−1^ Pa for 2 h. 

The specific surface area (A_BET_) was determined by the Brunauer–Emmett–Teller (BET) method. The external surface area (A_ext_) was determined by the comparative method of the t-plot, and the average statistical thickness t of the adsorption film was determined using the Harkins–Jura equation:

t = (n/n_m_) × σ, (2)

where n/n_m_ is the number of statistical monolayers in the film; n_m_ is the capacity of the monolayer in moles of the adsorbate; σ is the thickness of one layer, equal to 0.354 nm for nitrogen at 77 K, assuming the densest hexagonal packing of molecules in the adsorption film.

The analyzed isotherm was rebuilt into a t-plot as a function n = f(t). Replacing p/p_0_ with t made it possible to compare the isotherm and the standard t-curve. The external surface area (A_ext_) per unit mass of the solid was obtained as the difference between the specific surface, according to the BET equation, and the surface area of micropores (A_micro_), calculated by the t-method:

A_ext_ = A_BET_ − A_micro_, (3)

The pore volume (V_Des_) was calculated using the single point method, according to Gurvich [59,60]. The desorption cumulative volume (V_BJH Des_) of a group of pores with a diameter in the range from 1.7 to 300 nm was determined by the Barrett–Joyner–Halenda (BJH) method. The non-local density functional theory (NLDFT) model was used to describe the texture of the samples in terms of pore size distribution.

#### 4.2.2. Infrared Spectroscopy

FT-IR spectra were recorded on a Bruker Tensor-27 FT-IR spectrometer (Bruker Co., Billerica, MA, USA), in the range 4000–400 cm^−1^ and at a resolution of 4 cm^−1^, using tabletting of the powder with potassium bromide at a sample/KBr mass ratio of 2/800.

#### 4.2.3. X-ray Diffraction

X-ray phase analysis of the samples was carried out by X-ray diffraction on a DRON-3 diffractometer (Bourevestnik JSC., Saint Petersburg, Russia) using filtered Cu K_α_ radiation (λ = 0.15418 nm). The scanning step was 0.02 degrees and the scanning speed was 1 deg/min.

#### 4.2.4. Small-Angle Neutron Scattering

Small-angle neutron scattering measurements were performed on the Yellow Submarine instrument located at BNC in Budapest (Hungary) [61,62]. A collimation distance of 5 m and circular beam apertures of 25 and 7 mm in diameter defined the incoming beam divergence, while sample to detector distances of 1.2 and 5.5 m and neutron wavelengths of 0.63 nm defined the range of momentum transfer q = (4π/λ)sin(θ/2) = 0.06–3.1 nm^−1^. The scattered neutrons were detected by a two-dimensional position-sensitive BF_3_ gas detector. The raw data were corrected for sample transmissions and scattering of the empty cell, and converted to absolute units by comparison with the incoherent scattering of a 1 mm thick water sample. The powdered samples were filled into Hellma quartz cells of 2 mm flight path, and the measurements were performed at room temperature. The raw data were processed using the BerSANS software (version 14-Aug-2014) [63].

#### 4.2.5. Scanning Electron Microscopy

SEM images were taken on a TESCAN MIRA3 field emission scanning electron microscope by using the in-beam secondary electron detector with a 5 kV acceleration voltage. The samples were drop-casted on silicon surfaces from ethanolic suspension.

#### 4.2.6. Thermal Analysis

Thermal measurements were performed on a Setaram LabsysEvo (Setaram Instrumentation, Lyon, France) TG-DSC system in flowing high-purity nitrogen (99.999%; flow rate 60 mL/min) and separately in a synthetic air (20% O_2_ + 80% N_2_ ; flow rate 80 mL/min) atmosphere. Samples were weighed into 100 μL platinum crucibles (the reference cell was empty) and heated from 25 °C to 900 °C with a heating rate of 10 °C/min. The obtained data were blank corrected and further processed with the thermal analyzer’s processing software, Calisto Processing ver. 2.14 ( AKTS SA, Sierre, Switzerland). The thermal analyzer (both the temperature scale and calorimetric sensitivity) was calibrated by a multipoint calibration method, in which seven different certified reference materials were used to cover the thermal analyzer’s entire operating temperature range.

#### 4.2.7. Dye Sorption

The thiazine dye methylene blue (Sigma Aldrich, St. Louis, MO, USA) was taken as a model solution of organic pollutant. Sorption experiments for MB were conducted using procedures described previously [64]. The following experimental conditions were used: dose of adsorbent—2.0 g/L, concentration of the MB solution—0.5 mM (160 mg/L), initial pH of the MB solution—9.0, temperature—35 °C, and contact time—120 min. The pH of the one-component dye solution was adjusted to 9.0 by using 0.1 M NaOH. The sorption properties of the obtained sorbents were studied under static conditions at V/m = 0.5 L/g by using a thermostatic shaker ES 20/60 (BioSan Sia., Riga, Latvia) with a stirring speed of 200 rpm. Before sampling for analysis, the test solution was separated from the sorbents by using a Rotofix 32A laboratory centrifuge (Andreas Hettich GmbH & Co., Tuttlingen, Germany) at 5000 rpm for 5 min.

The effect of the dose of adsorbents (1.0–4.0 g/L) and the pH of the model pollutant solution (5.0–9.0) on the sorption characteristics was studied in a preliminary experiment.

Concentrations of MB in the supernatant were measured with a UV-vis spec**t**rophotometer SP-8001 (Metertech Inc., Taipei, Taiwan) at an adsorption wavelength λ_max_ = 664 nm. All sorption experiments were repeated at least 3 times to ensure accuracy of the obtained data. Sorption capacity of adsorbents based on nickel silicate (q_eq_, mg/g) and the removal efficiency of MB (α, %) were calculated according to Equations (4) and (5):

q_eq_ = (C_0_ − C_eq_) × V/m, (4)

α = (C_0_ − C_eq_) × 100/C_0_, (5)

where C_0_ (mg/L) is the initial dye concentration and C_eq_ (mg/L) is the dye concentration at equilibrium; V (L) is the dye solution volume and m (g) is the mass of adsorbent.

## Figures and Tables

**Table 1:** Specific surface area of nickel silicate samples calculated from nitrogen physical adsorption–desorption isotherms using Brunauer–Emmett–Teller (BET) equation; specific mesopore volume and average pore diameters calculated from desorption isotherms using Barrett–Joyner–Halenda (BJH) method and Gurvich’s 4V/A rule.

Sample Name	Ni/Si, %	pH	A_BET_, m^2^/g	V_BJH Des_, cm^3^/g	D_BJH Des_, nm	V_Des_, cm^3^/g	D_Des_ (4V/A), nm
pH3 00	0	3	1171	0.47	4.2	0.79	2.7
pH5 00	0	5	1081	0.58	4.7	0.81	3.0
pH8 00	0	8	971	0.47	5.7	0.72	3.0
pH5 05	5	3	1074	0.32	5.5	0.66	2.5
pH3 05	5	5	912	0.41	5.0	0.65	2.8
pH8 05	5	8	984	0.47	6.3	0.73	3.0
pH3 10	10	3	926	0.34	6.9	0.62	2.7
pH5 10	10	5	1032	0.39	4.1	0.71	2.8
pH8 10	10	8	999	0.44	4.7	0.70	2.8
pH3 15	15	3	876	0.39	6.7	0.65	3.0
pH5 15	15	5	947	0.81	4.8	0.91	3.8
pH8 15	15	8	1035	0.38	3.6	0.64	2.5
pH3 25	25	3	1198	0.25	5.1	0.64	2.2
pH5 25	25	5	1099	0.41	3.3	0.68	2.5
pH8 25	25	8	1126	0.46	3.9	0.72	2.6
pH3 50	50	3	1223	0.36	4.6	0.72	2.4
pH5 50	50	5	1195	0.38	3.7	0.72	2.4
pH8 50	50	8	1041	0.50	3.6	0.70	2.7

**Table 2:** Sorption capacity (q_eq_) and removal efficiency (α) of mesoporous nickel–silicate adsorbents for methylene blue.

	Sample		Sorption Characteristics	
Sample	Ni/Si, mol %	pH	q_eq_, mg/g	α, %
pH3 00	0/100	3	39.5	93.1
pH5 00	0/100	5	39.9	94.1
pH8 00	0/100	8	42.3	99.8
pH3 01	1/99	3	40.2	94.9
pH5 01	1/99	5	40.9	96.5
pH8 01	1/99	8	40.6	95.7
pH3 25	25/75	3	39.4	92.9
pH5 25	25/75	5	38.9	91.6
pH8 25	25/75	8	39.6	93.5
pH3 50	50/50	3	39.3	92.8
pH5 50	50/50	5	40.6	95.8
pH8 50	50/50	8	41.9	98.9

**Table 3:** Comparison of the sorption capacity (q_eq_) of sorbents based on mesoporous silica and/or metal silicates depending on the conditions of the sorption experiment.

Sorbent	Conditions	q_eq_, mg/g	Reference
Mesoporous silica MCM-41	m = 0.02 g (V/m = 2.5 L/g); C(MB) = 200 mg L^−1^; pH = 5.9; T = 30 °C; t = 180 min	48.0	[53]
Mesoporous silica MCM-41	m = 0.1 g (V/m = 0.5 L/g); C(MB) = 20 mg L^−1^; pH ~ 7; T = 21 °C; t = 30 h	9.4–9.9	[54]
Mesoporous silica MCM-41	m = 0.1 g (V/m = 0.5 L/g); C(MB) = 50 mg L^−1^; t = 24 h	24.5	[55]
Mesoporous silica Nb,Ta/MCM-41	m = 0.02 g (V/m = 1.0 L/g); C(MB) = 150 mg/L; pH = 10.0; T = 30 °C; t = 180 min	207.1	[28]
Mesoporous silica Al/MCM-41	m = 0.1 g (V/m = 1.0 L/g); C(MB) = 100 mg/L; pH = 8.0; T = 20 °C; t = 30 min	285.0	[56]
Mesoporous SBA-15	m = 0.01 g (V/m = 0.1 L/g); C(MB) = 40 mg/L; pH = 7.0; T = 25 °C; t = 20 min	351.0	[57]
Mesoporous SBA-15	C(MB) = 30 mg/L; pH = 9.0; m = 0.005 g; T = 20 °C; t = 40 min	223.0	[58]
Nickel silicates	m = 0.1 g (V/m = 0.5 L/g); C(MB) = 160 mg L^−1^; pH = 9.0; T = 35 °C; t = 120 min	38.9–42.3	This work

## Data Availability

The data presented in this study are available on request from the corresponding author.
